# University students’ views regarding gender in STEM studies: Design and validation of an instrument

**DOI:** 10.1007/s10639-022-11110-8

**Published:** 2022-05-28

**Authors:** Sonia Verdugo-Castro, Mª Cruz Sánchez-Gómez, Alicia García-Holgado

**Affiliations:** 1 GRIAL Research Group, Department of Didactics, Organization and Research Methods, Research Institute for Educational Sciences, Universidad de Salamanca, Salamanca, Spain; 2 GRIAL Research Group, Department of Didactics, Organization and Research Methods, Universidad de Salamanca, Salamanca, Spain; 3 GRIAL Research Group, Computer Science Department, Research Institute for Educational Sciences, Universidad de Salamanca, Salamanca, Spain

**Keywords:** STEM, Higher education, University, Validation, Questionnaire, Gender gap

## Abstract

Differences in the representation of diversity in higher education, emphasising the gender gap in some areas, are issues addressed from different research domains. Socially, gender roles have been constructed and are also related to professions. In this context, the Social Cognitive Career Theory explores the possible causes of segregation. This segregation is evident in Europe and Spain, as indicated by the European Institute for Gender Equality. This paper describes the design and validation process of an instrument to find out what opinions university students have about higher education studies in science, technology, engineering and mathematics (STEM), according to gender. After drafting the questionnaire, it was piloted in a non-experimental quantitative design in Spain. Subsequently, a validity and reliability study was applied to validate the items and construct their dimensionality. The process was implemented using Reliability Analysis and Exploratory Factor Analysis. Also, the dimensionality consists of five scales: Gender Ideology, Perception and Self-perception, Expectations about Science, Attitudes and Interests. Based on the results, it is concluded that the opinion about STEM studies is conditioned by personal elements, such as motivations, educational background and family and social influences, such as people who judged their decision, were their references or studied STEM programs. Finally, it is essential to pay socio-educational attention to the modulating components of decisions about which higher education studies to pursue. Awareness of the factors involved in the decision helps the educational community to establish mechanisms to prevent horizontal gender segregation. The instrument designed, validated and presented in this study provides a glimpse of possible causes for the gender gap in STEM higher education.

## Introduction

Figures reveal that science, technology, engineering and mathematics (STEM) sector suffers a loss of diversity once the higher stages of the career (Blickenstaff, 2005; Diekman et al., 2010; Sadler et al., 2012).

The latest figures updated by the European Institute for Gender Equality (EIGE) (2021) reveal that the representation of women in higher STEM studies in Europe, and specifically in Spain (ISCED levels 5–8), does not reach parity rates. They also show the extent of horizontal segregation in STEM higher education. In 2019, in Spain, 4.6% of the university student body represented male students of Information and Communication Technologies (ICT), while women accounted for 0.7%. In software and application development and analysis, 1.8% were men, while women accounted for 0.3%. Men accounted for 2.8% in electronics and automation, while women accounted for 0.4%. This is also the case in other European countries. For example, in ICT in Germany, 5.5% represented men, while women are only 1.5%. The same situation occurs in software and application development and analysis. For example, 5.6% were men in Estonia, while women represented only 2.2%. In Greece, 5.9% represented male students of electronics and automation, while women accounted for only 1.3%. This gender disparity is also observed in mathematics. For example, 0.4% represented male mathematics students in Ireland, while women accounted for only 0.1%. This apparent gender disparity in STEM fields across Europe also occurs in reverse in education and health (European Institute for Gender Equality (EIGE), 2021). However, it is striking that if the student body is not analysed by field, but in general, there is parity. For example, in 2019 in Spain, 53.7% of university students were women, and 46.3% were men. Therefore, the problem is not that more men than women are studying at university. In addition, Spanish universities do not discriminate based on gender when accessing their degrees.

In conclusion, the STEM education sector suffers from underrepresentation of gender diversity, mainly women Jacobs et al. (2017), and notably, this underrepresentation occurs in the engineering sector (Cvencek et al., 2021; Dou et al., 2020; Keku et al., 2021; Moote et al., 2020; Snyder et al., 2018).

Also, studies show that the origin of the gender gap is not biology, innate traits that might differentiate people according to their sex, or specific components of which professions people are to be engaged in according to their sex or gender (Bourdieu, 1984; Cheryan et al., 2013; Corbett & Hill, 2015; Nguyen & Ryan, 2008; Nguyen & Riegle-Crumb, 2021; O’Brien & Crandall, 2003). The origin of the gender gap lies in the social constructions forged in societies according to the interpretation of the world held by the people who compose them (Leslie et al., 2015; Master et al., 2016; Thébaud & Charles, 2018).

As Ertl et al. (2017) point out, in recent decades, the proportion of women in these fields has remained constant at approximately 25% in the European Union, thus not reaching parity representation. Also, as Talley and Martinez Ortiz (2017) point out, women account for less than 20% of engineering and computer science degrees, while they constitute less than 15% of all engineers working in the US.

There are false beliefs that women are more attracted to studies associated with caring for others or literary studies. At the same time, there is an erratic belief that men are attracted to more technical and rational professions of building and producing things. However, this justification is reductionist and binary (Diekman et al., 2010; Guo et al., 2018; Sikora & Pokropek, 2011; Su & Rounds, 2015). It seems that guilds are to be divided into two simplistic categories without considering the presence and importance of the environment.

Thus, the gender gap in STEM areas is a global problem and it is caused by different factors (Lent et al., 1994; Osborne et al., 2003). Different studies have been developed to investigate the influence of stereotypes on the decisions made concerning higher education studies (Cadaret et al., 2017; García-Holgado et al., 2019, 2020a, b; Makarova et al., 2019; Powell et al., 2012; Tomassini, 2021; Verdugo-Castro et al., 2019). The starting point is that different obstacles and barriers generate segregation in tertiary studies, knowing which ones can tackle them to reduce the gender gap.

The Social Cognitive Career Theory (Lent et al., 1994; Lent & Brown, 1996) is taken as a reference in the theoretical framework. The authors argue that internal and external factors condition the individual when acquiring knowledge and pursuing higher education. Thus, attention must be paid to the immediate context and the individual’s external experiences and influences. In addition, according to Bourdieu (1980), the existence of social representations as obstacles must be considered. The theories of these authors are baseline studies for successors, and several studies are currently being developed in this field. Recent research aim to reduce the Stereotype Threat and the Leaky Pipeline (Beasley & Fischer, 2012; Blickenstaff, 2005; Goulden et al., 2009).

Kang et al. (2019) emphasise the importance of preventing stereotyping and erratic patterns in teachers by focusing on future career prospects. The study results reveal that non-inclusive language, choice of heteronormative teaching material, and communication style can leave some of the student body out of context, particularly girls.

Stereotypes and prejudices formed and acquired early encourage gender discrimination. In this way, inequalities between men and women are reinforced throughout the different stages of their lives. In the academic sphere, textbooks’ content and hidden curriculum must be carefully considered since they are mechanisms for teaching. In some books, gender discrimination is transmitted through the representation of gender roles. As a result, students may come to think and act stereotypically concerning people depending on their gender because of the content they have been taught about. Regarding research and interventions in the field, Papadakis (2018) applies a qualitative study about the content of textbooks to identify sexist elements and gender stereotypes presented in materials used by computer science teachers and students in the general lyceum of Greece. Also, Papadakis et al. (2018) address female underrepresentation in the educational field of information science.

On the other hand, in the school context, Brauner et al. (2018) apply an initiative to enhance interest in STEM through robotics with German school children aged 10–13 years. In the initial phase, they are asked to draw a picture of a computer scientist. The results reveal social stereotypes about computer scientists, as they primarily draw men with a particular nerdy character and in solitary situations.

Further research on gender differences in the IT (Information Technology) sector is presented in Denmark (Borsotti, 2018). The study empirically investigates the main socio-cultural barriers to female participation in the Bachelor’s Degree in Software Development at Copenhagen’s University of Information Technology. The participants of the study attribute stereotypes as the reason for the gender gap.

On the other hand, gender stereotypes influence intrinsic factors, such as students’ self-concept. In the study by Ertl et al. (2017), the aim is to determine the academic self-concept of female university students studying a STEM-LPF degree, i.e. with a marked under-representation of women (equal to or less than 30% of women). The results of the interviews show the ambiguity of the family factor. In this study, all parents had STEM-related backgrounds, so they could support their daughters in the STEM field and stimulate their cognitive development. However, such support may also evoke an attribution of lower STEM skills. These attributions may influence their daughters’ academic self-concept in STEM.

Also, the study by Olmedo-Torre et al. (2018) analyses the influences on female STEM students. The authors divided women into two groups, those studying Computing, Communications, and Electrical and Electronic Engineering (CCEEE women) and those studying other non-CEEE degrees. The results reveal that the secondary school teachers and the peer group supported the women’s decision to study. However, their family did not support the decision at these levels. In addition, the CCEEE women showed less support from their family, teachers and peer group than the non-CCEEE women. This affected their self-concept, as the female CCEEEs felt less capable than their male peers at the beginning of their studies. Finally, on the causes they attributed to female under-representation in STEM, the responses were as follows: social stereotypes (31.5%), immediate environment (14.5%), women do not like engineering (11.03%), lack of information in high school (8.67%), stereotypes in education (8.18%), lack of female role models (7.93%), gender-biased toys (7.43%), job discrimination (7.19%) and engineering being difficult (5.82%).

According to García-Holgado et al. (2020a, b), the support received before starting the undergraduate course the support received before entering a STEM career is a determinant factor. The peers and family are the most important perceived supports, while teaching staff and institutional support have lower rates. Moreover, For female students, the support of their friends, schoolmates, and school matters.

Some authors take the research to the environment. For the study by Reich-Stiebert and Eyssel (2017), conducted in Germany, the aim was to explore the influence of gender stereotypes on learning with a robot in higher education. It was concluded that female participants outperformed male participants in typically male tasks and vice versa. Participants paid more attention to tasks that did not correspond to their gender and obtained better learning outcomes. Finally, the study by Finzel et al. (2018) also aimed to combat gender stereotypes that impact the gender gap by carrying out an intervention proposal to increase motivation for STEM studies. The intervention was conducted with students aged 16–18 within the “*make IT”* mentoring programme.

In this context, it should be stressed that different techniques and instruments can be used to understand the phenomenon of the gender gap and the causes of segregation. The various methodological options are designed for a specific population and with predefined objectives.

This study aims to determine how university students view higher education in science, technology, engineering, and mathematics according to gender, in order to acquired biases and design measures to remove them. For this reason, a specific instrument is required, aimed at university students.

Considering the optimisation of existing resources, instruments designed to address the gender gap in STEM programmes in higher education were analysed (Verdugo-Castro et al., 2019). After an extensive and exhaustive search in databases such as Web of Science, Scopus, ERIC, Dialnet and Google Scholar, 75 publications were identified that referred to questionnaires close to the study of the phenomenon (https://cutt.ly/bPKDRY). After reading the publications, only 18 indicated that the referenced instrument had undergone a validation process. Also, out of 18 possible resources, only 13 had actual proximity to the topic of study. However, there were two main reasons why it was decided that the instruments listed did not meet the study’s objective. The first reason was that, although the questionnaire was identified in the publication, it was not searchable in the databases, and the items were not available. The second cause was that the instruments analysed were not designed for the Spanish and European higher education framework, and were aimed at university students, focusing the research focus on opinion and bias detection. The instruments closest to the topic of the study were contextualised in the American and Chinese environments or focused exclusively on specific STEM domains, such as biology or computer science. They also target early childhood and adolescence and are not aimed at university ages.

No validated instruments dealing with the analysis of opinion on the topic of study were detected in the European context with university students. Therefore, the review results were revisited to identify data collection instruments associated with gender stereotypes and gender ideology. Those initially discarded as not fully aligned with all STEM disciplines but addressed bias and ideology were taken up.

Finally, five publications containing questionnaires that analysed gender ideology and stereotypes were identified (Banchefsky & Park, 2018; Duncan et al., 2019; Godwin, 2014; López Robledo, 2013; Rossi Cordero & Barajas Frutos, 2015) and could be used to construct a new instrument, the Questionnaire with university students on STEM studies in Higher Education (QSTEMHE).

None of the five instruments explicitly address the gender gap in STEM areas but are aimed at specific fields or target populations other than the main target of the research, which is the university population. The Banchefsky and Park (2018) instrument analyses gender ideologies, both negative and positive, and gender stereotypes concerning science. On the other hand, the Duncan et al. (2019) questionnaire delves into heteronormative attitudes and tolerance towards gender. On the other hand, the Godwin (2014) questionnaire is based on the Social Cognitive Theory of Career Development (Lent et al., 1994) and aims to study Critical Engineering Agency (CEA). The instrument addresses physical identity, mathematical identity, science identity, and agency beliefs. The López Robledo (2013) questionnaire analyses the attitude towards technology, the opinion about Information and Communication Technology disciplines, the obstacles that can be encountered in ICT studies, and the importance of having a reference or role model. Finally, in the Rossi Cordero and Barajas Frutos (2015) instrument, the categories of analysis are interests, values, and choice factors, for the individual dimension. The categories for the STEM studies dimension are motivation, role models and influences, and self-efficacy. Finally, there is a third dimension, challenges, and the categories for this are facilitators, obstacles, and opportunities.

For this reason, a selection of items was made to consider only those related to finding out what opinions university students have about higher studies in STEM, according to gender. The selection of the items was made by consensus by all the authors of this article, assessing their suitability for studying the gender gap in STEM, considering the theoretical framework.

Finally, the design process included a pilot phase in an exploratory study, and a validity and reliability study. After validation, the final version of the questionnaire was drafted, consisting of dimensionality of five scales: Gender Ideology, Perception and Self-perception, Expectations about Science, Attitudes and Interests.

The Questionnaire with university students on STEM studies in Higher Education (QSTEMHE) is a new instrument that helps researchers analyse university students’ perception about STEM studies to detect problems and provide new approaches to reduce the gender gap in these areas. To ensure the instrument’s validity and realiability, a methodological procedure of empirical validation has been followed. The procedures followed have been detailed to facilitate an understanding of the design of the questionnaire and its validation.

This paper has been divided into five parts. Section 2 describes the methodology applied to design and validate the instrument. Section 3 presents the data analysis and results. Section 4 discusses the results obtained. Finally, Section 5 summarizes the main conclusions of the work.

## Methods

**Fig. 1:**
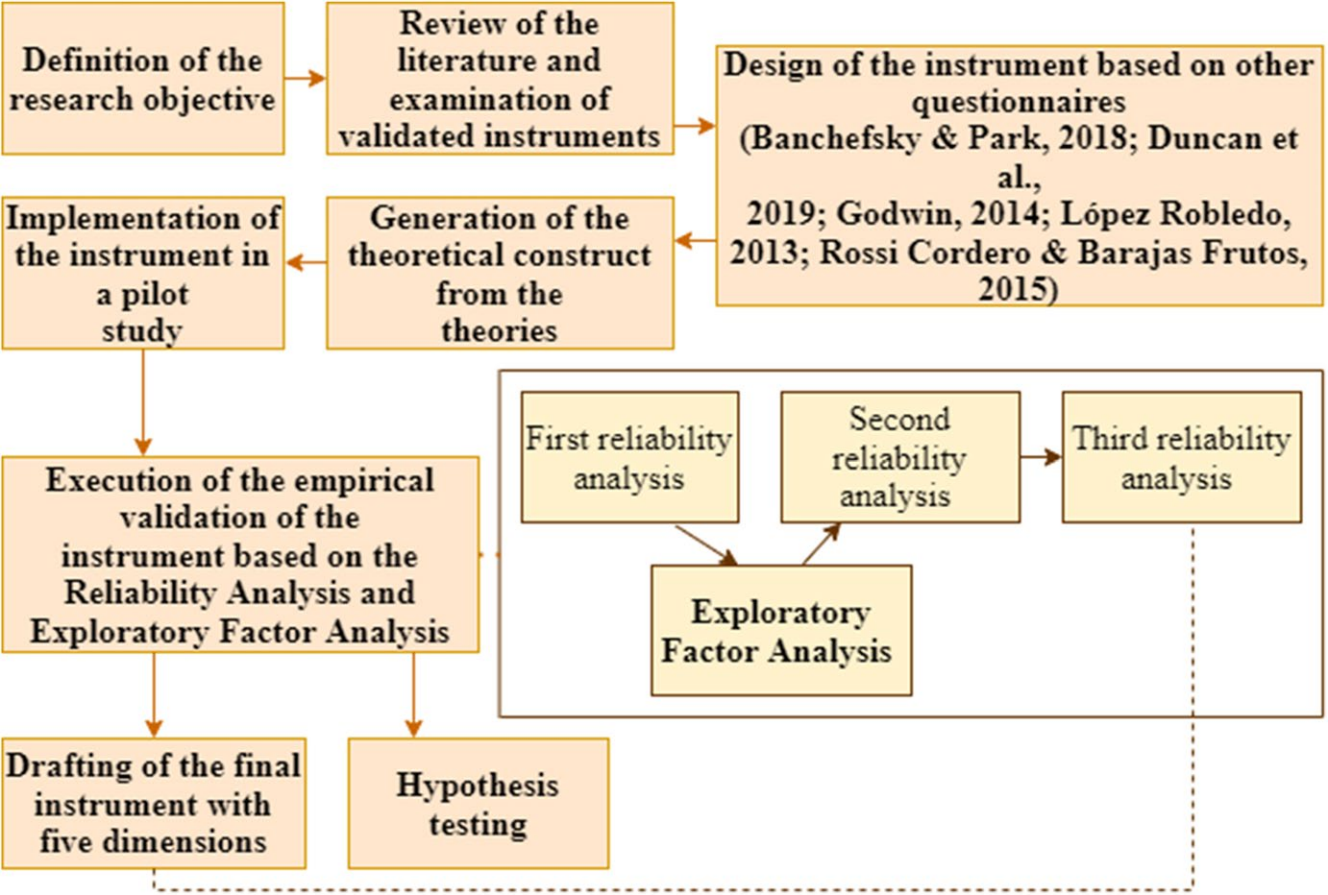
Workflow of study design and data analysis. Source: Own production

A non-experimental quantitative study (Sarrado et al., 2004) was applied to validate the QSTEMHE instrument. The instrument aims to find out the opinions of university students about higher studies in science, technology, engineering and mathematics, according to gender. The different phases that have been applied are identified in Fig. 1.

The empirical validation of the instrument was carried out through Reliability Analysis and Exploratory Factor Analysis. Once the theoretical construct was defined based on the authors of the sources that inspired the QSTEMHE, the statistical procedure was applied. Firstly, the first reliability analysis was applied to check the suitability of the theoretical construct. Once it was applied, it was found that the theoretical construct did not work at the empirical level. Therefore, Exploratory Factor Analysis was used to perform factor reduction. Once applied, a second reliability analysis was carried out. In this second analysis, an adaptation of the construct was introduced, respecting the principles of theoretical dimensionality. Finally, in the third reliability analysis, two scales were adjusted after checking the homogeneity of the items. Finally, the final version of the instrument has five dimensions. To conclude the statistical analysis procedure, hypothesis tests were conducted on the five scales with the predictor variables.

### Design of the instrument

The instrument’s items are based on previous questionnaires designed by other authors (Banchefsky & Park, 2018; Duncan et al., 2019; Godwin, 2014; López Robledo, 2013; Rossi Cordero & Barajas Frutos, 2015).

Concerning the present study, the topic of study addressed by QSTEMHE is the opinion that university students have about higher studies in science, technology, engineering and mathematics, according to gender.

The instrument was initially designed with 66 items (Verdugo-Castro et al., 2020): 37 ordinal items that have undergone a process of empirical validation (criterion variables), 5 open questions and 24 questions asking about socio-demographic variables (predictor variables).

First, before applying the validation process, seven dimensions were defined based on theory and the dimensions identified in the questionnaires of the selected items (Banchefsky & Park, 2018; Duncan et al., 2019; Godwin, 2014; López Robledo, 2013; Rossi Cordero & Barajas Frutos, 2015). These dimensions are Gender Ideology, Attitudes, Perceived Image, Interests, Women’s Skills, Perception and Self-Perception and Expectations about Science. These have been subjected to a statistical analysis process to validate the construct.

### Pilot study

The approval of the Bioethics Committee of the University of Salamanca (Spain) was obtained for this research before launching the pilot study. This procedure was necessary because the data collection was carried out with human subjects. Finally, a favourable report was obtained with registration number 557.

The exploratory study was conducted online in 2020 through an online application, a customised version of LimeSurvey. The data collection was done through email, sharing the questionnaire with mailing lists of professors from Spanish universities, using a snowball approach. During the data collection process, a limitation was encountered, namely the contingency caused by the COVID-19 health crisis. Initially, the pilot study was scheduled to be conducted face-to-face in classrooms, but this was impossible. The need to virtualise the teaching required the process to be carried out online.

### Study sample

The final sample consisted of 115 undergraduate students from Spanish universities (106 women, 8 men, and one person who does not identify as a man or a woman). The average age was 20, but the age range was from 18 to 34. There were 22 first-year students, 36 s-year students, 47 third-year students, 9 fourth-year students, and 1 fifth-year student. The participants came from Social and Legal Sciences (Pedagogy, Speech Therapy, Social Education), Health Sciences (Nursing and Pharmacy), Sciences (Chemistry), and Engineering and Architecture (Industrial Design Engineering). Finally, the participants were from eight different Spanish universities: University of Salamanca, Universitat de València, Universitat Politècnica de València, Universidad de Alcalá, Universidad Rovira i Virgili, Universidad Católica Santa Teresa de Jesús de Ávila, Universidad de Granada and Universidad de Sevilla.

## Data analysis and results

The analyses have been divided into two blocks. On the one hand, empirical validation was carried out with the ordinal items (criterion variables), as established in the protocol. Secondly, hypothesis tests were applied to the predictor variables (socio-demographic variables) based on the validated scales.

First, a statistical validation process was applied through an Exploratory Factor Analysis and three Reliability Analyses to study the dimensionality of the instrument and the validity and reliability of the items. Exploratory Factor Analysis is a data reduction technique that allows finding homogeneous groups of variables within several variables, i.e., dimensions (Akaike, 1987; Lloret-Segura et al., 2014; McCoach et al., 2013).

After applying the pilot study (Verdugo-Castro et al., 2020), the descriptive statistics of the mean and standard deviation of the items were calculated. Table 1 shows the results, where the instrument’s items are identified with their original enumeration from the questionnaire.

**Table 1:** Mean and standard deviation of ordinal items

Item	Source	Mean	SD
25. All humans are fundamentally the same, regardless of their gender.	(Banchefsky & Park, 2018)	3.50	0.949
26. If a woman decides to enter a traditionally masculine field, she will be more successful if she adopts the prevailing male customs and behaviours.		1.61	0.915
27. Men and women have different but equally useful ways of accomplishing tasks.		2.81	1.176
28. Having men and women work side-by-side increases the likelihood of conflict.		1.34	0.661
52. I feel restricted by the gender labels that people attach to me.	(Duncan et al., 2019)	2.13	1.056
53. I feel restricted by the expectations that people have of me because of my gender.		2.16	1.039
56. In the past, I have been teased or bullied for acting like the opposite sex.		1.62	0.942
31. Men who are not masculine are good role models.		1.82	1.418
32. Women who are not feminine are good role models.		1.78	1.407
29. Men should not act like women in the workplace.		1.86	1.407
30. Women should not act like men in the workplace.		1.88	1.434
54. In my childhood home, I was taught that men should act like men and women should act like women.		1.70	0.948
55. In the past, I have teased or bullied someone who dressed or acted like the opposite sex.		1.42	0.749
33. University studies are more important for men than for women.	(López Robledo, 2013)	1.16	0.410
34. Women must sacrifice their careers to support their children/family.		1.20	0.533
36. Women have the same technical skills as men.		3.31	1.012
37. In the IT field, a man’s performance will be better than a woman’s.		1.17	0.529
38. Women are capable of developing useful software.		3.66	0.887
40. Women and men have equal employment opportunities in ICT careers.		2.18	1.097
35. The well-being of the family is more important than the rewards of work.		2.63	1.095
58. I can enjoy a work environment mostly composed of men.		3.02	1.199
57. I feel comfortable working with people of the opposite gender.		3.61	0.757
39. At home, boys do more practical activities with their parents than girls (e.g. cars, tools, computers, etc.)	(Rossi Cordero & Barajas Frutos, 2015)	1.83	1.100
41. Boys prefer STEM-related hobbies.		1.86	0.954
46. Girls are not as interested as boys in STEM issues.		1.53	0.841
51. University studies in STEM are generally more attractive to boys.		2.28	1.039
42. There are more boys than girls in STEM studies as they are more freaks.		1.94	1.054
43. Women working in STEM areas have to be/act like men.		1.24	0.586
44. To have a successful career in STEM you need to think and act like a man.		1.17	0.517
47. STEM themes are more masculine than others.		1.48	0.776
50. STEM careers are not associated with the traditional role of women.		2.64	1.036
45. Girls are not as good as boys in STEM issues.		1.26	0.727
48. Girls have fewer natural abilities than men for STEM issues.		1.23	0.535
49. Most girls are better at other things (such as letters/languages) and choose studies in which they are better.		1.57	0.860
59. Science is helpful in my everyday life.	(Godwin, 2014)	3.19	0.999
60. Learning science has made me more critical in general.		2.83	1.184
61. Science and technologies will provide greater opportunities for future generations.		3.39	0.980

Based on the results, although there are low average values for the idea that if a woman decides to dedicate herself to a traditionally male field, she will have to adopt male customs and behaviours to be successful, the slightly positive trend for this statement is worrying. On the other hand, they also believe that men can act as they wish in the workplace without responding to stereotypical patterns. However, it is surprising that the variability is higher for the item referring to women. On the other hand, for item 42, the standard deviation is wide, so the variability leads us to think that some people do consider nerds to be those who study these subjects. Furthermore, while rejecting the idea that STEM subjects are more masculine than others, the standard deviation is higher. Finally, participants feel that men who do not conform to the male canon are not good role models. The results are similar for women. In addition, there is a belief that women and men have equal employment opportunities associated with ICT careers.

In addition, the correlations between the variables were calculated. Correlations indicate the strengths and directions established in a linear relationship and proportionality between two statistical variables. Pearson’s correlation was used to calculate the correlations. The correlations calculated through the Pearson Coefficient range from − 1 to 1. Values with a negative sign in the Pearson Coefficient reflect an inverse relationship, and values with a positive sign reflect a direct relationship. The value 0 reflects no relationship, and as a result approaches the values − 1 and 1, the relationship between the variables becomes stronger. Table 2 presents the high and medium-high correlations, which indicate a strong strength between the variables.

**Table 2:** Correlations between variables

High correlation		
29 with 30 (0.949)	31 with 32 (0.978)	52 with 53 (0.797)
Medium-high correlation		
43 with 44 (0.749)	52 with 56 (0.403)	59 with 60 (0.620)

As can be seen, the variables that have a high correlation between them are “Men should not act like women at work” (29) and “Women should not act like men at work” (30); “Men who are not masculine are good role models” (31) and “Women who are not feminine are good role models” (32); “I feel limited by the gender labels people put on me” (52) and “I feel limited by the expectations people have of me because of my gender” (53). On the other hand, the variables that have a medium-high correlation between them are “Women working in STEM areas have to be/act like men” (43) and “To have a successful career in STEM you need to think and act like a man” (44); “I feel restricted by the gender labels people put on me” (52) and “In the past, I have been teased or harassed for acting like the opposite sex” (56); and “Science is useful in my everyday life” (59) and “Learning science has made me more critical in general” (60). All these items will be included in the final version of the questionnaire.

### First reliability analysis

According to theory, based on the clustering of the items into the seven dimensions, the reliability of the instrument’s items was calculated. Table 3 presents the Cronbach’s alpha of the seven dimensions.

**Table 3:** Reliability statistics of the seven dimensions

Dimensions	Cronbach’s alpha	Number of items (N)
Gender Ideology scale (IG)	0.447	12
Attitude scale (AC)	0.390	7
Interests scale (INT)	-0.040	4
Perceived Image scale (IP)	0.372	5
Women’s Skills scale (HM)	0.471	3
Perception and Self-Perception scale (PAP)	0.760	3
Expectations about Science scale (EXC)	0.730	3

As can be seen from the calculation of Cronbach’s alpha, the statistical results are not adequate, meaning dimensionality will have to be studied again.

Also, item statistics for all dimensions are presented in Table 4.

**Table 4:** Item total statistics of the seven dimensions

Scale	Item	Corrected total item correlation	Cronbach’s alpha if the item is removed
Gender Ideology	25	0.163	0.426
	26	0.272	0.387
	27	0.030	0.472
	28	0.150	0.430
	31	0.222	0.405
	32	0.228	0.403
	33	0.188	0.429
	34	0.121	0.437
	36	0.154	0.429
	37	0.211	0.415
	38	0.195	0.419
	40	0.096	0.448
Attitude	29	0.596	− .015a
	30	0.519	0.062
	35	-0.007	0.442
	54	-0.035	0.442
	55	-0.074	0.441
	57	0.026	0.409
	58	0.104	0.392
Interests	39	0.030	− .118a
	41	0.000	− .063a
	46	0.054	− .151a
	51	-0.139	0.168
Perceived Image	42	0.176	0.339
	43	0.402	0.165
	44	0.365	0.188
	47	0.261	0.269
	50	-0.124	0.567
Women’s Skills	45	0.135	0.606
	48	0.421	0.168
	49	0.356	0.249
Perception and Self-Perception	52	0.752	0.474
	53	0.678	0.572
	56	0.378	0.887
Expectations about Science	59	0.600	0.591
	60	0.633	0.541
	61	0.445	0.759

Regarding the first reliability analysis of the Gender Ideology (IG) scale, we conclude that the items with low homogeneity are 27 and 40. For the Attitudes scale we conclude that the items with low homogeneity are 35, 54, 55, 57 and 58. For the Interests scale, the item with the lowest homogeneity is item 51. For the Perceived Image scale, the item with the lowest homogeneity is item 50. For the Women’s Skills scale, the item with the lowest homogeneity is item 45. For the Perception and Self-Perception scale, the item with the lowest homogeneity is item 56. For the Science Expectations scale, the item with the lowest homogeneity is 61.

### Exploratory factor analysis

Although seven dimensions are defined at the theoretical level, it has been decided to apply the Exploratory Factor Analysis because the dimensions constructed at the theoretical level, i.e., the dimensionality and the elements that compose it, report statistics with low values. This means that the variables do not behave homogeneously among themselves, which finally implies that the elements that at the theoretical level made up a dimension, in reality, do not make it up. It was also decided to apply Exploratory Factor Analysis because two of the dimensions proposed at the theoretical level, Women’s Skills (WM) and Perceived Image (PI), can be absorbed by other dimensions. The content they contain can, in turn, be compiled in other dimensions.

As for the lack of coincidence between the theoretical dimensionality and the results reported at the empirical level, as verified by the first reliability analysis, the reason is that the instrument measures people’s opinions on a topic of social impact such as gender. It is expected that there will be a diversity of opinion, which also produces more variability in the responses.

Therefore, we used the Exploratory Factor Analysis after applying the first Reliability Analysis. Initially and following the theory, we started with seven dimensions; however, the aim is to reduce these, obtaining those in which the items share meaning.

Before deciding to use Exploratory Factor Analysis for the study, it was necessary to apply the KMO and Bartlett’s test. The minimum recommended value of the KMO statistic is 0.5 to use the Exploratory Factor Analysis effectively. For the sample data of the study, the KMO test gives a significant value (Kaiser-Meyer-Olkin measure of sampling adequacy = 0.588), as can be seen in Table 5, so that the Exploratory Factor Analysis can be continued.

**Table 5:** KMO and Bartlett’s test

Kaiser-Meyer-Olkin measure of sampling adequacy		0.588
Bartlett’s test of sphericity	Approx. Chi-square	1871.590
	gl	666
	Sig.	< 0.001

Rotation is used to execute the Exploratory Factor Analysis, as it allows the variables to be plotted in a cloud. The Exploratory Factor Analysis carried out in this study was Principal Component Analysis with Oblimin Rotation. Using Principal Component Analysis as an extraction method, 13 different components were obtained.

To the total variance explained (Table 6), the total eigenvalues and the sum must be considered. The total variance represents how much variability is explained by the model. In this case, as can be seen, from the 13 components created through the principal component extraction method, 70% of the variability is explained. However, the aim is to reduce the dimensions. On the other hand, valuing the total of the eigenvalues, a value lower than 1 implies that it does not explain anything, so it must be higher than 1. In this case, the 13 components have a value higher than 1, although it can be seen that the components with the best values are the first 5, so the aim is to define five dimensions for the new dimensionality.

**Table 6:** Total variance explained

Total variance explained							
Cnt.	Initial eigenvalues			Sums of squared weights of extraction			Sums of squared rotation loadings
	Total	% of variance	% cumulative	Total	% of variance	% cumulative	Total
1	4.695	12.690	12.690	4.695	12.690	12.690	3.002
2	3.173	8.575	21.264	3.173	8.575	21.264	2.605
3	2.555	6.904	28.169	2.555	6.904	28.169	2.651
4	2.316	6.258	34.427	2.316	6.258	34.427	2.303
5	2.014	5.443	39.870	2.014	5.443	39.870	2.393
6	1.798	4.859	44.729	1.798	4.859	44.729	2.322
7	1.773	4.791	49.520	1.773	4.791	49.520	2.982
8	1.574	4.255	53.775	1.574	4.255	53.775	2.034
9	1.502	4.060	57.835	1.502	4.060	57.835	1.624
10	1.301	3.515	61.350	1.301	3.515	61.350	1.500
11	1.175	3.175	64.525	1.175	3.175	64.525	2.100
12	1.140	3.082	67.607	1.140	3.082	67.607	1.839
13	1.015	2.744	70.351	1.015	2.744	70.351	1.449
14	0.985	2.662	73.013				
15	0.937	2.531	75.545				
16	0.854	2.307	77.852				
17	0.830	2.243	80.095				
18	0.727	1.964	82.059				
19	0.673	1.820	83.879				
20	0.634	1.712	85.592				
21	0.613	1.655	87.247				
22	0.555	1.501	88.748				
23	0.545	1.472	90.220				
24	0.471	1.272	91.492				
25	0.461	1.245	92.737				
26	0.407	1.100	93.837				
27	0.388	1.049	94.886				
28	0.347	0.937	95.823				
29	0.334	0.902	96.725				
30	0.289	0.781	97.506				
31	0.237	0.640	98.146				
32	0.201	0.542	98.688				
33	0.167	0.452	99.140				
34	0.139	0.375	99.515				
35	0.127	0.344	99.860				
36	0.034	0.092	99.952				
37	0.018	0.048	100.000				

**Fig. 2:**
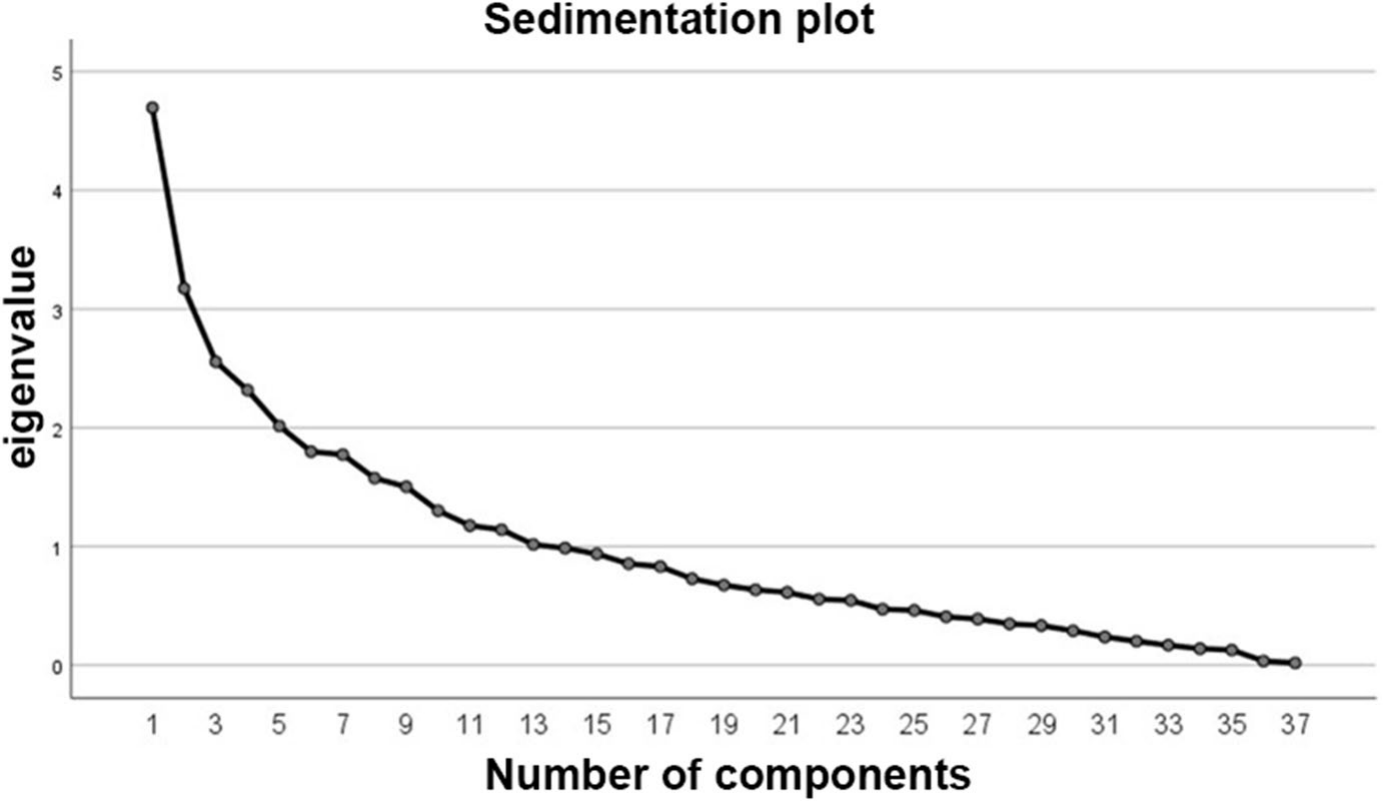
Sedimentation graph. Source: Own production with SPSS

In the sedimentation plot (Fig. 2), the information of the total eigenvalues of the total variance explained in the previous figure is visually represented.

The component matrix is also presented in Table 7. Although 13 different components have been formed, the variables do not saturate equally in all factors. Considering the results, eleven factors with high saturations would remain, but only three of the components have at least three items. Thus, it is found that factor reduction is required.

The variables attributed to its component are marked in bold for the proposal of the new dimensionality. The results obtained in the sedimentation graph have been considered for this composition. In the new dimensionality, coherence is sought between the variables and the dimensions, the smallest possible number of these and acceptable metric values for the new composition. In this way, the factorial reduction to 5 dimensions could be made. For this reason, in some variables, the box with the highest correlation appears in italics, given that although it is the correlation closest to 1 or -1, it is far from the main components, or there is no coherence with the other variables.

**Table 7:** Component matrix

Component matrix													
	Component												
	1	2	3	4	5	6	7	8	9	10	11	12	13
25	**0.361**	-0.188	0.02	-0.337	0.061	0.252	0.219	0.092	-0.032	0.11	-0.303	0.264	0.078
26	**0.48**	-0.133	-0.009	-0.056	0.067	0.049	0.206	0.071	-0.239	0.257	0.178	-0.191	0.173
27	-0.081	**-0.337**	0.162	-0.224	0.25	-0.032	-0.017	0.065	0.203	*0.385*	0.242	0.177	0.226
28	**0.328**	-0.196	0.146	0.259	0.148	0.121	0.204	0.292	-0.112	-0.198	-0.246	0.105	-0.169
31	-0.104	0.387	0.458	**0.328**	0.372	*-0.497*	0.091	0.05	-0.274	0.037	-0.003	0.11	0.01
32	-0.101	0.392	0.454	**0.347**	0.347	*-0.499*	0.092	0.045	-0.275	0.062	0.009	0.101	0.005
33	0.361	**0.437**	0.181	-0.176	0.203	0.167	-0.407	-0.037	0.071	-0.031	0.072	0.336	0.002
34	0.364	**0.37**	-0.052	-0.297	0.23	0.041	*-0.437*	0.142	0.017	-0.074	0.24	0.024	-0.142
36	**0.406**	-0.016	*-0.431*	-0.061	0.003	0.015	0.229	-0.149	-0.174	0.13	-0.067	0.335	0.173
37	**0.499**	0.019	-0.457	0.153	0.173	-0.014	0.094	-0.305	-0.207	0.003	0.003	0.16	-0.007
38	**0.296**	0.018	-0.026	-0.075	0.251	0.018	*0.42*	-0.243	0.147	0.27	-0.066	-0.047	-0.386
40	-0.025	**-0.342**	0.019	-0.064	*0.357*	0.19	0.028	0.302	-0.297	0.233	0.235	-0.31	0.1
29	0.253	0.181	0.103	**0.73**	-0.104	0.493	-0.042	0.139	0.064	0.05	-0.016	0.002	-0.033
30	0.198	0.152	0.109	**0.772**	-0.075	0.451	-0.07	0.083	0.116	0.059	-0.02	-0.024	-0.024
35	0.017	**-0.213**	0.218	-0.032	-0.207	-0.076	0.159	0.194	0.234	*-0.463*	0.233	0.279	0.343
54	-0.125	**0.408**	0.09	-0.229	-0.237	0.332	0.183	0.346	-0.203	0.014	0.006	0.19	0.13
55	-0.261	**0.447**	-0.079	-0.081	-0.131	0.138	-0.073	0.237	-0.104	0.273	-0.222	0.079	0.057
57	0.339	-0.32	0.09	-0.14	**0.496**	-0.007	0.167	0.158	0.234	-0.048	0.052	0.097	-0.278
58	0.423	-0.353	0.018	0.064	**0.486**	0.054	0.006	0.277	0.263	-0.094	-0.221	-0.126	0.165
39	-0.111	0.137	-0.173	0.322	**0.311**	0.036	-0.231	-0.145	*0.404*	0.328	0.123	0.034	0.237
41	**0.48**	-0.162	-0.061	-0.08	-0.074	-0.027	0.047	0.137	0.054	-0.128	0.038	-0.353	0.017
46	**0.524**	0.017	-0.425	0.127	0.06	-0.358	-0.136	0.159	0.012	-0.172	-0.235	0.025	0.147
51	-0.35	0.163	-0.017	-0.016	**0.401**	0.288	0.19	-0.256	-0.034	-0.109	0.061	-0.101	*0.446*
42	**0.464**	0.029	0.042	0.004	-0.351	-0.232	-0.31	0.182	0.057	0.294	0.134	-0.293	0.009
43	**0.482**	0.302	0.385	-0.243	0.133	0.158	-0.237	-0.313	0.01	-0.208	0.006	-0.095	0.008
44	**0.519**	0.265	0.378	-0.201	0.166	0.272	-0.102	-0.262	-0.135	-0.218	-0.052	-0.222	0.028
47	**0.515**	0.025	-0.144	0.139	-0.084	-0.286	-0.174	0.192	0.122	-0.091	0.271	0.125	0.061
50	-0.163	**0.275**	-0.281	0.159	0.081	-0.085	0.193	-0.358	*0.477*	-0.142	-0.035	-0.119	0.068
45	**0.355**	0.007	-0.302	-0.02	0.044	-0.146	-0.405	0.054	-0.007	0.189	*-0.452*	0.048	0.14
48	**0.498**	0.14	-0.275	0.174	-0.146	0.015	0.176	-0.284	-0.257	0.13	0.291	0.049	0.041
49	**0.522**	0.015	-0.296	0.092	-0.052	0.047	0.117	0.013	-0.276	-0.232	0.265	0.017	-0.012
52	0.063	**0.65**	-0.292	-0.115	0.089	-0.03	0.281	0.327	0.258	-0.021	0.088	-0.075	-0.123
53	-0.048	**0.678**	-0.243	-0.188	0.083	0.052	0.311	0.324	0.218	0.049	0.148	0	-0.056
56	-0.02	**0.513**	-0.16	-0.151	0.005	-0.165	0.172	0.042	-0.091	-0.095	-0.257	-0.378	0.216
59	*0.473*	-0.029	**0.308**	0.055	-0.39	-0.221	0.306	-0.124	0.262	0.116	0.027	0.012	-0.018
60	0.512	0.034	**0.544**	0.065	-0.204	-0.097	0.285	0.028	0.136	0.105	-0.121	-0.06	0.271
61	*0.406*	0.217	**0.379**	-0.311	-0.226	0.036	0.019	-0.17	0.139	0.244	-0.086	0.079	-0.005

**Fig. 3:**
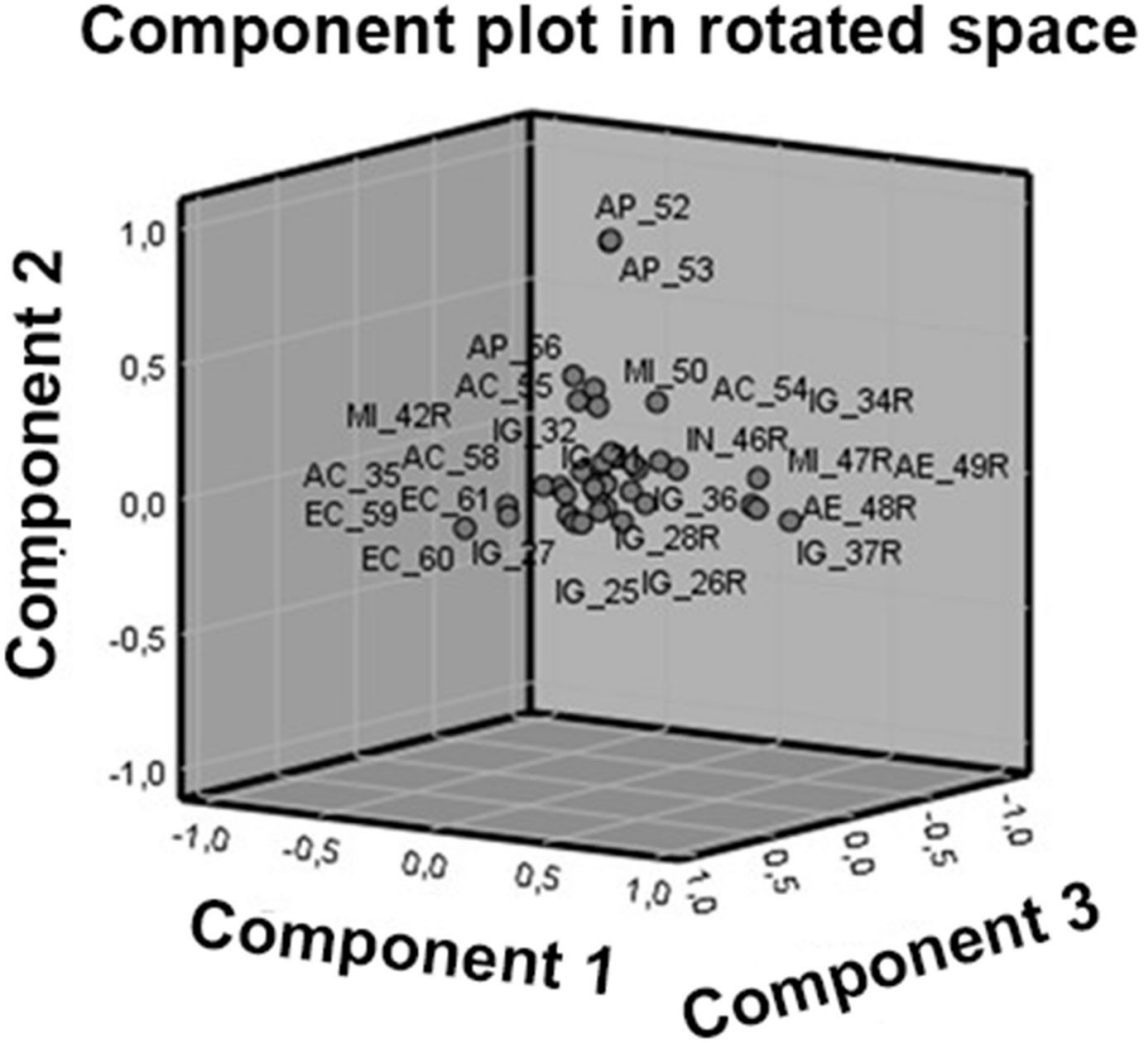
The component graph in rotated space. Source: Own production with SPSS

Finally, Fig. 3 shows the component graph in rotated space, where the closeness or distance between the different items grouped can be seen.

### Second reliability analysis

The five new scales or dimensions were formulated once the Exploratory Factor Analysis was carried out. The dimensions of Women’s Skills and Perceived Image would have been absorbed by the dimensions of Gender Ideology (Women’s Skills), Interests and Attitudes (Perceived Image). The Gender Ideology, Attitudes, Interests, Perception and Self-Perception, and Expectations about Science scales have been maintained. For this reason, the Reliability Analysis has been applied again, based on the reformulation of the dimensionality, to find out the behaviour of the items in the new dimensions and group them into these.

Table 8 presents the Cronbach’s alpha of the five dimensions.

**Table 8:** Reliability statistics of the five dimensions

Dimensions	Cronbach’s alpha	Number of items (N)
Gender Ideology scale (IG)	0.608	15
Attitude scale (AC)	0.745	4
Interests scale (INT)	0.334	4
Perception and Self-Perception scale (PAP)	0.354	11
Expectations about Science scale (EXC)	0.730	3

As can be seen from the results obtained, the reliability results have improved considerably after the construct check. The scales that could still improve their Cronbach’s alpha are the gender ideology, interests, and perception and self-perception scales. For this reason, it is advisable to study which items show low homogeneity.

Also, item statistics for the dimensions are presented in Table 9.

**Table 9:** Item total statistics of the five dimensions

Scale	Item	Scale mean if the element has been suppressed	Scale variance if the item is suppressed	Total correlation of corrected items	Cronbach’s alpha if the item has been dropped
Gender Ideology	25	24.37	23.289	-0.175	0.667
	26	26.26	20.124	0.198	0.599
	28	26.53	20.321	0.307	0.583
	36	24.56	23.056	-0.154	0.669
	37	26.70	21.228	0.222	0.596
	38	24.21	21.693	0.013	0.631
	41	26.01	19.237	0.292	0.581
	42	25.93	17.574	0.442	0.547
	43	26.63	19.762	0.476	0.564
	44	26.70	20.407	0.409	0.576
	45	26.61	19.977	0.321	0.579
	46	26.34	18.665	0.443	0.555
	47	26.39	18.591	0.507	0.546
	48	26.63	20.094	0.460	0.569
	49	26.30	19.389	0.325	0.576
Perception and Self-perception	27	18.83	14.016	-0.099	0.440
	33	20.49	13.796	0.186	0.331
	34	20.44	13.688	0.141	0.334
	35	19.01	13.903	-0.070	0.419
	40	19.46	14.444	-0.134	0.446
	50	19.00	13.211	0.033	0.373
	52	19.51	10.305	0.459	0.169
	53	19.49	10.182	0.493	0.154
	54	19.95	12.190	0.218	0.295
	55	20.23	13.001	0.179	0.317
	56	20.03	11.885	0.271	0.273
Expectations about Science	59	6.23	3.352	0.600	0.591
	60	6.58	2.684	0.633	0.541
	61	6.03	3.868	0.445	0.759
Attitudes	29	5.48	11.076	0.546	0.681
	30	5.46	11.058	0.531	0.690
	31	5.52	10.953	0.555	0.676
	32	5.56	11.284	0.520	0.696
Interests	39	8.90	4.035	0.112	0.351
	51	8.46	4.286	0.087	0.373
	57	7.13	4.448	0.224	0.243
	58	7.72	3.010	0.310	0.073

The second reliability analysis of the Gender Ideology scale concludes that the items with low homogeneity are 25 (All humans are fundamentally the same, regardless of their gender) and 36 (Women have the same technical skills as men). It was decided to eliminate these two items, reducing the scale from 15 items to 13 to improve the scale’s reliability. For the Perception and Self-perception scale we concluded that the items with low homogeneity are 27 (Men and women have different but equally useful ways of accomplishing tasks), 35 (The well-being of the family is more important than the rewards of work) and 40 (Women and men have equal employment opportunities in ICT careers). It was decided to eliminate these three items, reducing the scale from 11 items to 8 to improve the scale’s reliability.

Before the exclusion of the items, the initial theoretical composition of the dimensionality was reviewed based on the theory of the instruments that inspired the questionnaire (Banchefsky & Park, 2018; Duncan et al., 2019; Godwin, 2014; López Robledo, 2013; Rossi Cordero & Barajas Frutos, 2015) to verify that the deleted items did not constitute fundamental components of the theoretical dimensionality. Item 25 and 35 do not contain content directly linked to the gender gap in STEM but are cross-cutting content. Item 27 can be applied in STEM but also other sectors. Finally, the content of item 40 can be analysed through other items that have been consolidated in the questionnaire.

Also, there are no items with low homogeneity for the Science Expectations, Interests and Attitudes scales, so it is decided not to remove items.

Finally, suppose it is reduced from 37 items to 32. In that case, a new Reliability Analysis must be applied for the dimensions that suffer reductions: the Gender Ideology scale and the Perception and Self-perception scale.

### Third reliability analysis

The third Reliability Analysis was applied to the dimensions of Gender Ideology and Perception and Self-perception. These are the ones for which items were eliminated, namely items 25, 27, 35, 36 and 40. For the dimensions of Expectations about Science, Attitudes, and Interests, the results of the second Reliability Analysis were maintained. The Cronbach’s alpha of the dimensions addressed has improved by eliminating the five items with low homogeneity.

Table 10 presents the Cronbach’s alpha of the dimensions.

**Table 10:** Reliability statistics of the dimensions

Dimensions	Cronbach’s alpha	Number of items (N)
Gender Ideology scale (IG)	**0.733**	13
Perception and Self-Perception scale (PAP)	**0.646**	8

Cronbach’Square cross-tabulationss Alpha values are represented in bold

As can be seen, by removing the five items, the results for Cronbach’s alpha have improved substantially for both scales.

Also, item statistics for the dimensions are presented in Table 11.

**Table 11:** Item total statistics of the seven dimensions

Scale	Item	Scale mean if the element has been suppressed	Scale variance if the item is suppressed	Total correlation of corrected items	Cronbach’s alpha if the item has been dropped
Gender Ideology	26	19.45	21.724	0.196	0.739
	28	19.72	21.676	0.345	0.719
	37	19.90	22.568	0.276	0.726
	38	17.40	23.909	-0.053	0.769
	41	19.20	20.144	0.371	0.716
	42	19.12	18.810	0.472	0.701
	43	19.82	21.098	0.518	0.704
	44	19.89	21.715	0.467	0.711
	45	19.80	21.477	0.331	0.720
	46	19.53	19.532	0.537	0.693
	47	19.58	19.807	0.553	0.693
	48	19.83	21.461	0.501	0.707
	49	19.50	20.480	0.387	0.713
Perception and Self-perception	33	12.86	13.226	0.185	0.646
	34	12.82	13.308	0.091	0.660
	50	11.37	11.587	0.182	0.664
	52	11.89	8.978	0.609	0.523
	53	11.86	8.910	0.638	0.514
	54	12.32	11.273	0.279	0.632
	55	12.60	11.909	0.286	0.627
	56	12.40	10.558	0.409	0.594

Table 12 shows the Cronbach’s alpha values resulting from the third reliability analysis for each scale (dimension).

**Table 12:** Reliability statistics for the five new dimensions of the instrument

Dimension	Cronbach’s alpha
Gender Ideology (IG)	0.733
Perception and Self-Perception (PAP)	0.646
Expectations about science (EXC)	0.730
Attitudes (AC)	0.745
Interests (INT)	0.334

Finally, Table 13 shows the composition of the items in the final dimensionality

**Table 13:** Final dimensions with their items

Component 1: Gender Ideology (IG) Total items: 13	26. If a woman decides to enter a traditionally masculine field, she will be more successful if she adopts the prevailing male customs and behaviours.
	28. Having men and women work side-by-side increases the likelihood of conflict.
	37. In the IT field, a man’s performance will be better than a woman’s.
	38. Women are capable of developing useful software.
	41. Boys prefer STEM-related hobbies.
	42. There are more boys than girls in STEM studies as they are more freaks.
	43. Women working in STEM areas have to be/act like men.
	44. To have a successful career in STEM you need to think and act like a man.
	45. Girls are not as good as boys in STEM issues.
	46. Girls are not as interested as boys in STEM issues.
	47. STEM themes are more masculine than others.
	48. Girls have fewer natural abilities than men for STEM issues.
	49. Most girls are better at other things (such as letters/languages) and choose studies in which they are better.
Component 2: Perception and Self-Perception (PAP) Total items: 8	33. University studies are more important for men than for women.
	34. Women must sacrifice their careers to support their children/family.
	50. STEM careers are not associated with the traditional role of women.
	52. I feel restricted by the gender labels that people attach to me.
	53. I feel restricted by the expectations that people have of me because of my gender.
	54. In my childhood home, I was taught that men should act like men and women should act like women.
	55. In the past, I have teased or bullied someone who dressed or acted like the opposite sex.
	56. In the past, I have been teased or bullied for acting like the opposite sex.
Component 3: Expectations about Science (EXC) Total items: 3	59. Science is helpful in my everyday life.
	60. Learning science has made me more critical in general.
	61. Science and technologies will provide greater opportunities for future generations.
Component 4: Attitudes (AC) Total items: 4	29. Men should not act like women in the workplace.
	30. Women should not act like men in the workplace
	31. Men who are not masculine are good role models.
	32. Women who are not feminine are good role models.
Component 5: Interests (INT) Total items: 4	39. At home, boys do more practical activities with their parents than girls (e.g. cars, tools, computers, etc.)
	51. University studies in STEM are generally more attractive to boys.
	57. I feel comfortable working with people of the opposite gender.
	58. I can enjoy a work environment mostly composed of men.

### Definition of dimensions

Gender ideology is related to the social conception of gender roles and patterns. It may be marked by philosophies of equal opportunity and inclusion of gender diversity, or it may be characterised by binary logics based on masculinity and femininity, understood as canons to be followed (Keller, 1995). On the other hand, misperceptions about careers in STEM domains significantly impede women’s ability to pursue STEM career paths (Diekman et al., 2010). In turn, self-perception may also lead to low-interest rates and enrolment or continuation. Expectations about science relate to the outcomes that are expected from science, as well as from the study of science. Outcome expectations are beliefs about the effects of doing certain activities (Lent et al., 1994), in this case, about studying STEM domains or not.

Furthermore, attitudes towards science, according to Osborne et al. (2003), can be understood as the feelings, beliefs and values that a person has about an object, which may be, in this case, science, science at school, the impact science has on society, the science-based labour market, including scientists themselves. Finally, in terms of interest, there are studies such as that of Blázquez et al. (2011), which investigated the interest of students in Spain in pursuing higher education in engineering. The results reveal that 30% of the participants in the pilot study are not at the right age, i.e. ready to start higher education, which means that some of them decide which studies to pursue without being qualified to do so. Therefore, education systems aim to encourage interest in STEM fields; however, there is a loss of interest among students, resulting in declining enrollment. Studies such as Blickenstaff (2005) and Sadler et al. (2012), among others, show that women tend toward health and social sciences, while men tend towards technical and exact sciences; which suggests that the enjoyment of the subjects and the student body’s interest need to be deepened.

### Post-validation analysis of the instrument

#### Frequencies, descriptive statistics and correlations of the new dimensions of the instrument

After validation, the statistics for the five dimensions were calculated and are shown in Table 14.

**Table 14:** Descriptive statistics of the dimensions

Scale	Nº	Minimum	Maximum	Mean		Standard Devi.
	Sta.	Sta.	Sta.	Sta.	Error deviation	Sta.
IG	115	1.00	2.90	1.6965	0.03583	0.38422
PAP	115	1.00	2.75	1.7937	0.04279	0.45888
EXC	114	2.00	4.00	3.3611	0.05253	0.56090
AC	106	1.00	4.00	2.3325	0.09934	1.02279
INT	115	1.25	4.00	2.9065	0.04792	0.51384
Scale	Variance	Skewness		Kurtosis		
	Sta.	Sta.	Error deviation	Sta.	Error deviation	
IG	0.148	0.940	0.226	0.745	0.447	
PAP	0.211	-0.013	0.226	-0.857	0.447	
EXC	0.315	-0.513	0.226	-0.573	0.449	
AC	1.046	0.162	0.235	-1.158	0.465	
INT	0.264	-0.163	0.226	0.318	0.447	

#### Correlations

In turn, Pearson correlations were calculated for the scales to study the degree to which the scores are associated, i.e., the relationship established between the scales.

As a result, it is concluded that the correlations between the five scales are low and medium-low (Table 15), which is positive because each scale addresses a different element to be measured within the instrument.

**Table 15:** Correlations for the scales

		IG	PAP	EXC	AC	INT	
IG	Pearson correlation	1	0.213*	− 0.349**	0.281**	0.295**	
	Sig. (bilateral)		0.022	0	0.003	0.001	
	N	115	115	114	106	115	
PAP	Pearson correlation	0.213*	1	-0.033	0.12	0.017	
	Sig. (bilateral)	0.022		0.729	0.22	0.856	
	N	115	115	114	106	115	
EXC	Pearson correlation	− 0.349**	-0.033	1	-0.071	-0.027	
	Sig. (bilateral)	0	0.729		0.47	0.772	
	N	114	114	114	105	114	
AC	Pearson correlation	0.281**	0.12	-0.071	1	0.156	
	Sig. (bilateral)	0.003	0.22	0.47		0.111	
	N	106	106	105	106	106	
INT	Pearson correlation	0.295**	0.017	-0.027	0.156	1	
	Sig. (bilateral)	0.001	0.856	0.772	0.111		
	N	115	115	114	106	115	
Note: * means that the correlation is significant at the 0.05 level (bilateral)							
Note: ** means that the correlation is significant at the 0.01 level (bilateral)							

#### Sample distribution

The Kolmogorov-Smirnov normality test (Table 16) has also been applied to the five scales to see what the sample distribution is like. The p-value result (bilateral asymptotic sig.) for the five scales is less than 0.05, which means significant differences for the sample.

**Table 16:** Kolmogorov-Smirnov test for one sample

	IG	PAP	EXC	AC	INT
N	115	115	114	106	115
Test statistic	0,140	0.091	0.171	0.140	0.111
Asymptotic sig.(bilateral)	,000	0.021	0.000	0.000	0.001

#### Chi-Square cross-tabulations

It is necessary to apply a test, in this case, a non-parametric test, to check whether or not there are significant differences in response to an item according to several groups (two or more).

As the items were ordinal, the Pearson Chi-square test was used. Contingency tables were used for the application. Besides Pearson’s Chi-square, Kendall’s Tau-b and Kendall’s Tau-c tests (ordinal by ordinal) were also applied for this analysis.

The socio-demographic data used for the comparison were gender, the area in which they live, the branch of university studies they studied, their motivation for choosing their studies, the position of their studies in the selection process for university entrance, previous interest in STEM-related higher education studies, participation in any STEM initiative or activity before university studies, whether a family member or other person in their environment has studied STEM, has been their academic reference, or has judged their educational decision, the socio-economic level to which consider the area in which they grew up to belong, and the studies of their parents.

About the Pearson’s Chi-square results, of the 32 final instrument items, 28 show significant differences according to some socio-demographic data. The item with the most differences is item 56, with 11 characteristics.

#### Hypothesis testing for the dimensions

Finally, different hypothesis tests were applied for the five dimensions (criterion variables) and the instrument’s socio-demographic variables (predictor variables). The hypothesis tests were also based on non-parametric methods. The Mann Whitney U test was used for two independent groups, and three independent groups or more groups, the Kruskal-Wallis test was applied.

In total, 18 significant differences were found by socio-demographic variables regarding the five dimensions. The results are shown in Table 17.

**Table 17:** Results of the significant differences of the hypothesis tests by dimensions

Dimension	Cronbach’s alpha
Significant differences on the Gender Ideology scale	Area - sig. 0.027
	Motivation to choose studies (option ‘Possibility to work on projects’) - sig. 0.035
	Field of study (Health and Social Sciences, and Engineering and Chemistry) - sig. 0.032
	Previous interest in STEM - sig. 0.035
Significant differences in the Perception and Self-perception scale	Socio-economic and cultural status - sig. 0.016
	Motivation for study choice (option ‘It is an option to travel’) - sig. 0.010
	Preference in choice of studies (first choice, second choice or another choice) - sig. 0.048
	Family and environment that questioned their decision (option ‘A teacher’) - sig. 0.047
Significant differences on the scale of Expectations about science	Area - sig. 0.020
	Course - sig. 0.004
	Motivation for choosing studies (option ‘Possibility to work on projects’) - sig. 0.005
	Field of study (Social and Health Sciences, and Engineering and Chemistry) - sig. 0.001
	Vocational Education and Training - sig. 0.034
	Previous interest in STEM - sig. < 0.001
	Age (18–19, 20–21, 22–34) - sig. 0.039
Significant differences on the Attitudes scale	Education level of parent/legal guardian - sig. 0.044
Significant differences on the Interests scale	Environment has studied STEM (option ‘Mother’) - sig. 0.033
	Family and role model/ role model environment for studies (option ‘Other relative (uncle, cousin, grandfather, etc.)’) - sig. 0.041

## Discussion

Interpreting the results obtained for the sample in the hypothesis tests for all five scales, some of the factors that modulate the results for the Gender Ideology dimension are the environment in which the person lives, the motivations for choosing higher education, whether they have previously shown interest in the STEM domains and the higher education they are pursuing. The results are consistent with the Social Cognitive Career Theory (Lent et al., 1994; Lent & Brown, 1996), which shows that career choice is made according to intrinsic and extrinsic and environmental factors that modulate the decision. Furthermore, Bourdieu (1984) also states that human beings are involved in social development, which conditions how they think and act and how they decide.

For the sample studied, living in a rural environment is a possible cause of predisposition to gender stereotyping, which could be explained by the traditional culture in which specific social patterns are still ingrained (Thébaud & Charles, 2018). On the other hand, choosing higher education because of the motivation to work on projects significantly decreases the predisposition to gender stereotypes. In this sense, authors such as Finzel et al. (2018) advocate the work of motivation and interests to close the gender gap in STEM studies. At the same time, those who have participated in the pilot experience and who have shown an interest in STEM domains before their university studies show lower average values for gender stereotyping bias. Also, another author who advocates working on interests to reduce horizontal segregation in education is Brauner et al. (2018), who fights to eradicate the gap through initiatives. However, this contrasts with the studies finally pursued, as participants studying engineering and chemistry disciplines have higher rates of stereotyping than those studying social sciences and health sciences, as also revealed by Borsotti (2018).

On the scale of Perception and Self-perception, those in the sample who perceive the area in which they have grown up as having a medium-low or medium socio-economic and cultural level have higher rates of stereotyping. This result aligns with Bourdieu’s (1980) theory of social representation based on social capital. Continuing with the motivations, choosing to study because of the desire to travel helps to reduce stereotyping rates. Also, those who decided their studies first have lower rates than those who chose their studies second or further away. All these findings align with Brauner et al. (2018) theories. Finally, careful attention should be paid to the communication between teachers and students since students who feel judged by a male teacher are predisposed to higher rates of erratic perceptions and self-perceptions. In this direction, Papadakis (2018) and Papadakis et al. (2018) advocate prevention from the educational spheres and also the care of the hidden curriculum.

On the Science Expectations scale, for the sample, those living in urban settings, followed by those living in intermediate neighbourhoods and finally in rural settings, have higher expectations about science. Also, people in their first year and younger have higher expectations. Again, the motivation to work on projects reveals favourable results. People who have chosen their studies for this reason and those who showed interest in STEM before higher education have higher expectations of science. In this sense, authors such as Brauner et al. (2018), Lent et al. (1994), Lent & Brown (1996), and Osborne et al. (2003) are known to argue that horizontal segregation is induced by factors extrinsic to the individual, but also intrinsic, such as the individual’s educational background. For all these reasons, it is essential to work based on motivations. On the other hand, people who have no vocational training and those studying social sciences and health sciences have higher expectations than those who have vocational training or are studying engineering and chemistry disciplines.

Concerning the Attitudes scale, it is observed that the father’s education is related to the results obtained, with lower weights as the studies are more advanced. Finally, for the Interests scale, it is again observed that the parents’ studies influence. In this case, the mother’s education modulates towards higher weights. Studying the educational environment and the family and social environment is essential. Knowing which people in their environment have studied STEM fields, which people have been their references or have judged their decision, makes it possible to establish the networks of influences on choosing higher education (Lent et al., 1994; Lent & Brown, 1996).

As is evident, the factors that condition the decision on which higher education studies to pursue and the elements that intervene in the opinion on these options according to gender are different (Blickenstaff, 2005; Lent et al., 1994). Personal, academic, family and social factors are part of the construct that modulates the presence or absence of bias (Blázquez et al., 2011; Lent et al., 1994). Knowing the direction of these elements, preventive and corrective measures can be applied from a socio-educational perspective (Lent & Brown, 1996).

Finally, initiatives are a valuable mechanism to respond to the gender gap in STEM higher education from a gender equality perspective. They represent strategies through which interests and motivations can be directed and strengthened. This idea is further supported by authors consulted in the literature, such as Blickenstaff (2005), Cadaret et al. (2017), Cheryan et al. (2013), Corbett & Hill (2015), Diekman et al. (2010), and Ertl et al. (2017). Strategies for recruiting women and girls into STEM studies include mentoring, tutoring and modelling.

## Conclusions

The study presented in this article arose from the need to design a questionnaire to study the opinion of university students on STEM studies according to gender. Being able to analyse stereotypes and biases is relevant to addressing the gender gap in educational settings, following the guidelines of some authors such as Sadler et al. (2012). This is important because horizontal segregation leads to a loss of diversity in STEM higher education, as Jacobs et al. (2017), Moote et al. (2020), and Snyder et al. (2018) point out. After a methodological procedure for designing and validating an instrument, gender stereotypes in science, technology, engineering, and mathematics can be identified. The instrument has been named Questionnaire with university students on STEM studies in Higher Education (QSTEMHE).

The results obtained from the application of the instrument favour the design of preventive and direct interventions. Some interventions can be directly applied in classrooms or campaigns to bring STEM areas closer to the student population (García Peñalvo et al., 2019).

The results of the study also reveal and confirm what was expected. The family, social and peer group environment, and educational references condition the decision on which studies to pursue. Moreover, the perception and opinion about STEM studies in higher education are also conditioned by internal elements such as motivations, interests, attitudes, self-confidence, self-efficacy, etc. Thus, the two theories on which this research is based, social cognitive career theory (Lent et al., 1994; Lent & Brown, 1996) and Bourdieu’s theory (1980), are confirmed.

Finally, some important limitations need to be considered. First, the health situation due to the pandemic caused by COVID-19 slowed down the data collection process, as classes were virtualised, and it was impossible to go to the classrooms in person. The online data collection required several reminders via e-mail; however, it is hoped that the study will be replicated in the future with a higher sample size to confirm the results obtained. Another limitation found was the reluctance of some people to answer the questionnaire, stating that they did not find it sufficiently interesting as it was a gender study. This perceived difficulty could be studied in depth in the future, given that we also consider it a result. Finally, we assume that the conditions of confinement and, therefore, the impossibility of being able to go to the classrooms in person, together with the reluctance of some people to respond, has meant that the volume of responses from men has been lower than that of responses from women. The application of the final version of the instrument on a larger sample has been planned when the restrictions due to the health crisis have been relaxed. To this end, it is hoped to extend the questionnaire to students from all Spanish universities, achieving representative groups and equality between the different gender groups. Another future line of action is to be able to extend the questionnaire to other European environments, including adapting the contextual questions for non-European foreign countries where its application can be studied.

## Data Availability

The datasets generated and/or analysed during this study are not publicly available because the data are part of an unfinished doctoral thesis but are available from the corresponding author upon reasonable request.

## Abbreviations

AC: Attitudes scale.
CCEEE: Computing, Communications, and Electrical and Electronic Engineering.
CNT: Component.
EXC: Expectations about Science scale.
IG: Gender Ideology scale.
INT: Interests scale.
PAP: Perception and Self-Perception scale.
IP: Perceived Image scale.
STEM: Science, Technology, Engineering, Mathematics.
STEM-LPF: STEM subjects with a low proportion of women.
HM: Women’s Skills scale.

## References

[CR1] Akaike H (1987). Factor analysis and AIC. Psychometrika.

[CR2] Banchefsky S, Park B (2018). Negative gender ideologies and gender-science stereotypes are more pervasive in male-dominated academic disciplines. Social Sciences.

[CR3] Beasley MA, Fischer MJ (2012). Why they leave: The impact of stereotype threat on the attrition of women and minorities from science, math and engineering majors. Social Psychology of Education.

[CR4] Blázquez, M., Castro, M., Tovar, E., Llamas, M., Plaza, I., & Meier, R. (2011). Are engineering students decreasing? A Spanish case study. *2011 IEEE Global Engineering Education Conference (EDUCON)*, 242–251. 10.1109/EDUCON.2011.5773144

[CR5] Blickenstaff JC (2005). Women and science careers: Leaky pipeline or gender filter?. Gender and Education.

[CR6] Borsotti, V. (2018). Barriers to gender diversity in software development education: Actionable insights from a danish case study. *40th ACM/IEEE International Conference on Software Engineering: Software Engineering Education and Training, ICSE-SEET 2018; Gothenburg; Sweden; 30 May 2018 through 1 June 2018*, 146–152. 10.1145/3183377.3183390

[CR7] Bourdieu P (1980). Le capital social. Actes de la recherche en sciences sociales.

[CR8] Bourdieu, P. (1984). Ethos, habitus, hexis. *Questions de sociologie* (pp. 1–2). Les Éditions de Minuit

[CR9] Brauner, P., Ziefle, M., Schroeder, U., Leonhardt, T., Bergner, N., & Ziegler, B. (2018). Gender Influences On School Students’ Mental Models of Computer Science A Quantitative Rich Picture Analysis with Sixth Graders. *Proceedings of the 4th Conference on Gender & IT (Genderit ’18)*, 113–122. 10.1145/3196839.3196857

[CR10] Cadaret MC, Hartung PJ, Subich LM, Weigold IK (2017). Stereotype threat as a barrier to women entering engineering careers. Journal of Vocational Behavior.

[CR11] Cheryan S, Plaut VC, Handron C, Hudson L (2013). The stereotypical computer scientist: Gendered media representations as a barrier to inclusion for women. Sex Roles.

[CR12] Corbett, C., & Hill, C. (2015). *Solving the equation: The variables for women’s success in engineering and computing*. AAUW

[CR13] Cvencek D, Brečić R, Gaćeša D, Meltzoff AN (2021). Development of math attitudes and math self-concepts: Gender differences, implicit–explicit dissociations, and relations to math achievement. Child Development.

[CR14] Diekman AB, Brown ER, Johnston AM, Clark EK (2010). Seeking congruity between goals and roles: a new look at why women opt out of science, technology, engineering, and mathematics careers. Psychological Science.

[CR15] Dou, R., Bhutta, K., Ross, M., Kramer, L., & Thamotharan, V. (2020). The effects of computer science stereotypes and interest on middle school boys’ career intentions. *ACM Transactions on Computing Education*, *20*(3). 10.1145/3394964

[CR16] Duncan, S. G., Aguilar, G., Jensen, C. G., & Magnusson, B. M. (2019). Survey of heteronormative attitudes and tolerance toward gender non-conformity in mountain west undergraduate students. *Frontiers in Psychology*, *10*. 10.3389/fpsyg.2019.00793 10.3389/fpsyg.2019.00793 PMC6470281 31031673

[CR17] Ertl, B., Luttenberger, S., & Paechter, M. (2017). The Impact of gender stereotypes on the self-concept of female students in STEM subjects with an under-representation of females. *Frontiers in Psychology*, *8*(703). 10.3389/fpsyg.2017.00703 10.3389/fpsyg.2017.00703 PMC5434750 28567022

[CR18] European Institute for Gender Equality (EIGE). (2021). *Browse Gender Statistics | Gender Statistics Database*. European Institute for Gender Equality. https://eige.europa.eu/gender-statistics/dgs

[CR19] Finzel, B., Deininger, H., & Schmid, U. (2018). From beliefs to intention: Mentoring as an approach to motivate female high school students to enrol in computer science studies. *4th Conference on Gender and IT, GenderIT 2018; Heilbronn UniversityHeilbronn; Germany; 14 May 2018 through 15 May 2018*, 251–260. 10.1145/3196839.3196879

[CR20] García-Holgado A, González-González CS, Peixoto A (2020). A comparative study on the support in engineering courses: A case study in Brazil and Spain. IEEE Access: Practical Innovations, Open Solutions.

[CR21] García-Holgado A, Verdugo-Castro S, González C, Sánchez-Gómez MC, García-Peñalvo FJ (2020). European proposals to work in the gender gap in STEM: A systematic analysis. Revista Iberoamericana de Tecnologias del Aprendizaje.

[CR22] García-Holgado, A., Vázquez-Ingelmo, A., Verdugo-Castro, S., González, C. S., Sánchez-Gómez, M. C., & García-Peñalvo, F. J. (2019). Actions to promote diversity in engineering studies: A case study in a Computer Science Degree. En *2019 IEEE Global Engineering Education Conference (EDUCON), (9–11 April 2019, Dubai, UAE)*. IEEE. 10.1109/EDUCON.2019.8725134

[CR23] García-Peñalvo, F. J., Bello, A., Domínguez, A., & Romero Chacón, R. M. (2019). Gender Balance Actions, Policies and Strategies for STEM: Results from a World Café Conversation. *Education in the Knowledge Society*, *20*. 10.14201/eks2019_20_a3

[CR24] Godwin, A. (2014). Understanding female engineering enrollment: Explaining choice with critical engineering agency. *All Dissertations*. https://tigerprints.clemson.edu/all_dissertations/1787

[CR25] Goulden, M., Frasch, K., & Mason, M. A. (2009). *Patching America’s leaky pipeline in the sciences*. Center for American Progress. Berkeley Law University of California, 52

[CR26] Guo, J., Eccles, J. S., Sortheix, F. M., & Salmela-Aro, K. (2018). Gendered pathways toward STEM careers: The incremental roles of work value profiles above academic task values. *Frontiers in Psychology*, *9*. 10.3389/fpsyg.2018.01111 10.3389/fpsyg.2018.01111 PMC6050506 30050478

[CR27] Jacobs, J. A., Ahmad, S., & Sax, L. J. (2017). Planning a career in engineering: Parental effects on sons and daughters. *Social Sciences*, *6*(2), 10.3390/socsci6010002

[CR28] Kang J, Hense J, Scheersoi A, Keinonen T (2019). Gender study on the relationships between science interest and future career perspectives. International Journal of Science Education.

[CR29] Keku, D., Paige, F., Shealy, T., & Godwin, A. (2021). Recognizing differences in underrepresented civil engineering students’ career satisfaction expectations and college experiences. *Journal of Management in Engineering*, *37*(4), 10.1061/(ASCE)ME.1943-5479.0000902

[CR30] Keller, E. F. (1995). *Reflections on Gender and Science*. Yale University Press

[CR31] Lent RW, Brown SD (1996). Social cognitive approach to career development: An overview. The Career Development Quarterly.

[CR32] Lent RW, Brown SD, Hackett G (1994). Toward a unifying social cognitive theory of career and academic interest, choice, and performance. Journal of Vocational Behavior.

[CR33] Leslie SJ, Cimpian A, Meyer M, Freeland E (2015). Expectations of brilliance underlie gender distributions across academic disciplines. Science.

[CR34] Lloret-Segura S, Ferreres-Traver A, Hernández-Baeza A, Tomás-Marco I (2014). El Análisis Factorial Exploratorio de los Ítems: Una guía práctica, revisada y actualizada. Anales de Psicología.

[CR35] López Robledo, D. M. (2013). *El género como factor determinante al escoger una carrera profesional en sistemas de información*. Universidad del Turabo. Escuela de Negocios y Empresarismo

[CR36] Makarova, E., Aeschlimann, B., & Herzog, W. (2019). The gender gap in STEM fields: The impact of the gender stereotype of math and science on secondary students’ career aspirations [Original research]. *Frontiers in Education*, *4*(60). 10.3389/feduc.2019.00060

[CR37] Master A, Cheryan S, Meltzoff AN (2016). Computing whether she belongs: Stereotypes undermine girls’ interest and sense of belonging in computer science. Journal of Educational Psychology.

[CR38] McCoach, D. B., Gable, R. K., & Madura, J. P. (2013). *Instrument development in the affective domain: School and corporate applications, 3rd ed* (pp.xvi, 307). Springer Science + Business Media. 10.1007/978-1-4614-7135-6

[CR39] Moote J, Archer L, DeWitt J, MacLeod E (2020). Comparing students’ engineering and science aspirations from age 10 to 16: Investigating the role of gender, ethnicity, cultural capital, and attitudinal factors. Journal of Engineering Education.

[CR40] Nguyen HHD, Ryan AM (2008). Does stereotype threat affect test performance of minorities and women? A meta-analysis of experimental evidence. The Journal of Applied Psychology.

[CR41] Nguyen, U., & Riegle-Crumb, C. (2021). Who is a scientist? The relationship between counter-stereotypical beliefs about scientists and the STEM major intentions of Black and Latinx male and female students. *International Journal of STEM Education*, *8*(1). 10.1186/s40594-021-00288-x 10.1186/s40594-021-00288-x PMC10857866 38343634

[CR42] O’Brien LT, Crandall CS (2003). Stereotype threat and arousal: Effects on women’s math performance. Personality and Social Psychology Bulletin.

[CR43] Olmedo-Torre N, Sanchez Carracedo F, Salan Ballesteros MN, Lopez D, Perez-Poch A, Lopez-Beltran M (2018). Do female motives for enrolling vary according to STEM profile?. IEEE Transactions on Education.

[CR44] Osborne J, Simon S, Collins S (2003). Attitudes towards science: A review of the literature and its implications. International Journal of Science Education.

[CR45] Papadakis S (2018). Gender stereotypes in Greek computer science school textbooks. International Journal of Teaching and Case Studies.

[CR46] Papadakis S, Tousia C, Polychronaki K (2018). Women in computer science. The case study of the Computer Science Department of the University of Crete, Greece. International Journal of Teaching and Case Studies.

[CR47] Powell A, Dainty A, Bagilhole B (2012). Gender stereotypes among women engineering and technology students in the UK: lessons from career choice narratives. European Journal of Engineering Education.

[CR48] Reich-Stiebert, N., & Eyssel, F. (2017). (Ir)relevance of Gender?: On the Influence of Gender Stereotypes on Learning with a Robot. *ACM/IEEE International Conference on Human-Robot Interaction*, *Part F127194*, 166–176. 10.1145/2909824.3020242

[CR49] Rossi Cordero, A. E., & Barajas Frutos, M. (2015). Elección de estudios CTIM y desequilibrios de género. *Enseñanza de las ciencias*, 0059–0076. 10.5565/rev/ensciencias.1481

[CR50] Sadler PM, Sonnert G, Hazari Z, Tai R (2012). Stability and volatility of STEM career interest in high school: A gender study. Science Education.

[CR51] Sarrado JJ, Cléries X, Ferrer M, Kronfly E (2004). Evidencia científica en medicina: ¿única alternativa?. Gaceta Sanitaria.

[CR52] Sikora, J., & Pokropek, A. (2011). *Gendered Career Expectations of Students: Perspectives from PISA 2006. OECD Education Working Papers No. 57*. OECD Publishing

[CR53] Snyder, T. D., de Brey, C., & Dillow, S. A. (2018). Digest of Education Statistics, 2016.Ies National Center for Education Estatistics,970

[CR54] Su, R., & Rounds, J. (2015). All STEM fields are not created equal: People and things interests explain gender disparities across STEM fields. *Frontiers in Psychology*, *6*(189), 10.3389/fpsyg.2015.00189 10.3389/fpsyg.2015.00189 PMC4340183 25762964

[CR55] Talley, K. G., & Martínez Ortiz, A. (2017). Women’s interest development and motivations to persist as college students in STEM: A mixed methods analysis of views and voices from a Hispanic-Serving Institution. *International Journal of STEM Education*, *4*(1). 10.1186/s40594-017-0059-2

[CR56] Thébaud S, Charles M (2018). Segregation, stereotypes, and STEM. Social Sciences.

[CR57] Tomassini C (2021). Gender gaps in science: Systematic review of the main explanations and the research agenda. Education in the Knowledge Society.

[CR58] Verdugo-Castro, S., García-Holgado, A., & Sánchez-Gómez, M. C. (2019). Analysis of instruments focused on gender gap in STEM education. In M. Á. Conde-González, F. J. Rodríguez Sedano, C. Fernández Llamas, & F. J. García-Peñalvo (Eds.), *Proceedings of the 7th International Conference on Technological Ecosystems for Enhancing Multiculturality (TEEM 2019) (León, Spain, October 16–18, 2019)* (pp. 999–1006). ACM. 10.1145/3362789.3362922

[CR59] Verdugo-Castro, S., Sánchez-Gómez, M. C., García-Holgado, A., & Bakieva, M. (2020). Pilot study on university students’ opinion about STEM studies at higher education. In F. J. García-Peñalvo (Ed.), *Proceedings of the Eight International Conference on Technological Ecosystems for Enhancing Multiculturality (TEEM 2020) (Salamanca, Spain, October 21–23, 2020)* (pp. 158–165). ACM

